# Gene Expression Profiling and Chromatin Immunoprecipitation Identify DBN1, SETMAR and HIG2 as Direct Targets of SOX11 in Mantle Cell Lymphoma

**DOI:** 10.1371/journal.pone.0014085

**Published:** 2010-11-22

**Authors:** Xiao Wang, Stefan Björklund, Agata M. Wasik, Alf Grandien, Patrik Andersson, Eva Kimby, Karin Dahlman-Wright, Chunyan Zhao, Birger Christensson, Birgitta Sander

**Affiliations:** 1 Department of Laboratory Medicine, Division of Pathology, Karolinska Institutet and Karolinska University Hospital Huddinge, Stockholm, Sweden; 2 Center for Infectious Medicine and Center for Experimental Hematology, Karolinska Institutet, Stockholm, Sweden; 3 Department of Medicine, Division of Hematology, Karolinska Institutet and Karolinska University Hospital, Stockholm, Sweden; 4 Department of Hematology, Stockholm South Hospital, Stockholm, Sweden; 5 Department of Biosciences and Nutrition, Novum, Karolinska Institutet, Stockholm, Sweden; University of Barcelona, Spain

## Abstract

The SRY (sex determining region Y)-box 11 (SOX11) gene, located on chromosome 2p25, encodes for a transcription factor that is involved in tissue remodeling during embryogenesis and is crucial for neurogenesis. The role for SOX11 in hematopoiesis has not yet been defined. Two genes under direct control of SOX11 are the class- III β-tubulin gene (TUBB3) in neural cells and the transcription factor TEA domain family member 2 (TEAD2) in neural and mesenchymal progenitor cells. Normal, mature lymphocytes lack SOX11 but express SOX4, another member of the same group of SOX transcription factors. We and others recently identified SOX11 as aberrantly expressed in mantle cell lymphoma (MCL). Since SOX11 is variably expressed in MCL it may not be essential for tumorigenesis, but may carry prognostic information. Currently, no specific functional effects have been linked to SOX11 expression in MCL and it is not known which genes are under influence of SOX11 in lymphoma. In this study we found variable expression of SOX11, SOX4 and SOX12 mRNA in mantle cell lymphoma cell lines. Downregulation of SOX11 expression by siRNA verified that SOX11 controlled the expression of the gene TUBB3 in the MCL cell line Granta 519. Furthermore we identified, by global gene expression analysis, 26 new target genes influenced by siRNA SOX11 downmodulation. Among these genes, DBN1, SETMAR and HIG2 were found to be significantly correlated to SOX11 expression in two cohorts of primary mantle cell lymphomas. Chromatin immunoprecipitation (ChIP) analysis showed that these genes are direct targets of the SOX11 protein. In spite of almost complete downregulation of the SOX11 protein no significant effects on Granta 519 cell proliferation or survival in short term *in vitro* experiments was found. In summary we have identified a number of genes influenced by SOX11 expression in MCL cell lines and primary MCL. Among these genes, DBN1, SETMAR and HIG2 are direct transcriptional targets of the SOX11 protein.

## Introduction

The SRY (sex determining region Y)-box 11 (SOX11) transcription factor was recently discovered as a new marker in mantle cell lymphoma (MCL), expressed in both cyclin D1 positive and negative cases [1], [2], [3], [4]. SOX11, located on chromosome 2p25, is a member of the SOX gene family and was discovered in 1995 [5]. Approximately 20 SOX genes have been identified and they are divided into eight subgroups according to the degree of similarity within and outside the high mobility domain (HMG) [6]. All SOX genes have unique and specific expression patterns and they control cell survival, proliferation and differentiation in numerous processes during embryogenesis (reviewed in [7]). SOX11, together with SOX4 and SOX12, belongs to the SOXC group, sharing a high degree of homology both in the HMG domain and the C-terminal transactivation domain [8]. In the mouse, Sox11 is important for organ development, neurogenesis, neural cell survival and neurite outgrowth [8], [9], [10] [11] while Sox4 is crucial for B lymphocyte differentiation [12]. Upregulated SOX11 has been found in brain tumors and in ovarian carcinoma, where it has been associated to tumorigenesis and clinical outcome [13], [14], [15], [16], [17]. The recently reported aberrant expression of SOX11 in most MCL [1], [2], [3], [4], [18], [19] raises the hypothesis that SOX11 may have a role in the pathogenesis of MCL.

MCL is characterized by enhanced cell proliferation, impaired cell death pathways and reduced response to DNA damaging agents and is therefore difficult to treat [20]. These features can only be partly explained by the t(11;14)(q13;q32) causing dysregulation of cyclin D1 expression. While the t(11;14) is an early event in the pathogenesis of most MCL other genetic events are necessary for lymphoma development [21], [22].

The class-III β-tubulin gene (TUBB3) is up-regulated by Sox11 in neural cells [8]. Using siRNA-mediated knockdown of SOX11 in the MCL cell lines Granta 519 and JeKo, we found that TUBB3 was influenced by SOX11 also in MCL. Additionally, 26 genes were significantly downregulated upon SOX11 silencing in Granta 519. Among these genes, drebrin 1 (DBN1), SET domain and mariner transposase fusion gene (SETMAR) and hypoxia-inducible protein 2 (HIG2) expression correlated to SOX11 expression in a series of SOX11 positive MCL [4]. We found a strong correlation between SOX11 and DBN1, SETMAR and HIG2 expression in a recently published study on SOX11 positive and negative MCL [19]. Furthermore, in MCL cell lines and primary MCL cells, DBN1 significantly correlated to SOX11 expression.

## Results

### Expression of SOXC Transcription Factors in MCL Cell Lines – High Expression of SOX11 in Granta 519

**Figure 1:**
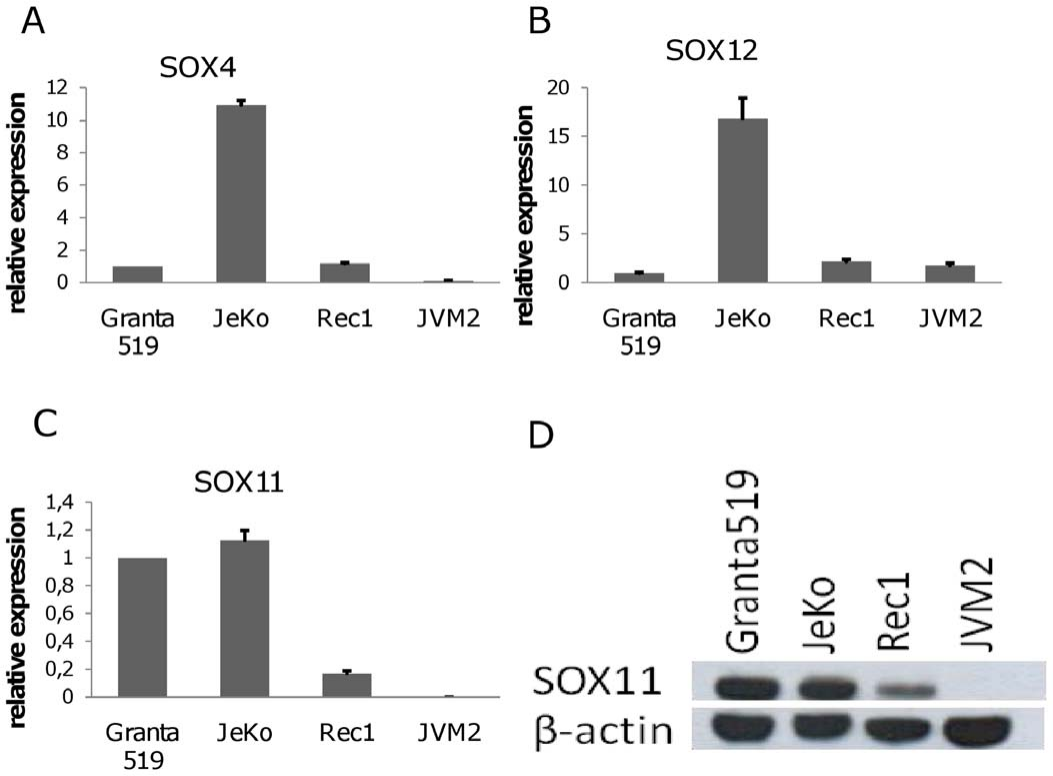
Expression of SOXC genes in MCL cell lines. Expression of SOX4 (A), SOX12 (B) and SOX11 (C) in the MCL cell lines Granta 519, JeKo, Rec1 and JVM2 by quantitative RT- PCR. Granta 519 express high levels of SOX11 mRNA but low levels of the other SOXC genes while no SOX11 mRNA expression was detected in JVM2. (D) WB showed that Granta 519, JeKo and Rec1, but not JVM2, express SOX11 protein.

SOX4, SOX11 and SOX12 may have partially overlapping effects on transcriptional regulation [8]. We therefore investigated the expression of the three SOXC transcription factors, SOX4, SOX11 and SOX12 in the t(11;14) positive MCL cell lines Granta 519, Rec1, JeKo and JVM-2 (Figure 1). Granta 519, Rec1 and JeKo expressed SOX11 mRNA and protein while JVM-2 was negative for SOX11 expression (Figure 1). SOX4 and SOX12 protein expression could not be measured due to lack of commercially available specific antibodies. The Granta 519 cell line expressed high levels of SOX11 and very little SOX4 and SOX12 and was therefore chosen for further studies.

### Silencing of SOX11 in Granta 519 Modifies TUBB3 Expression

**Figure 2:**
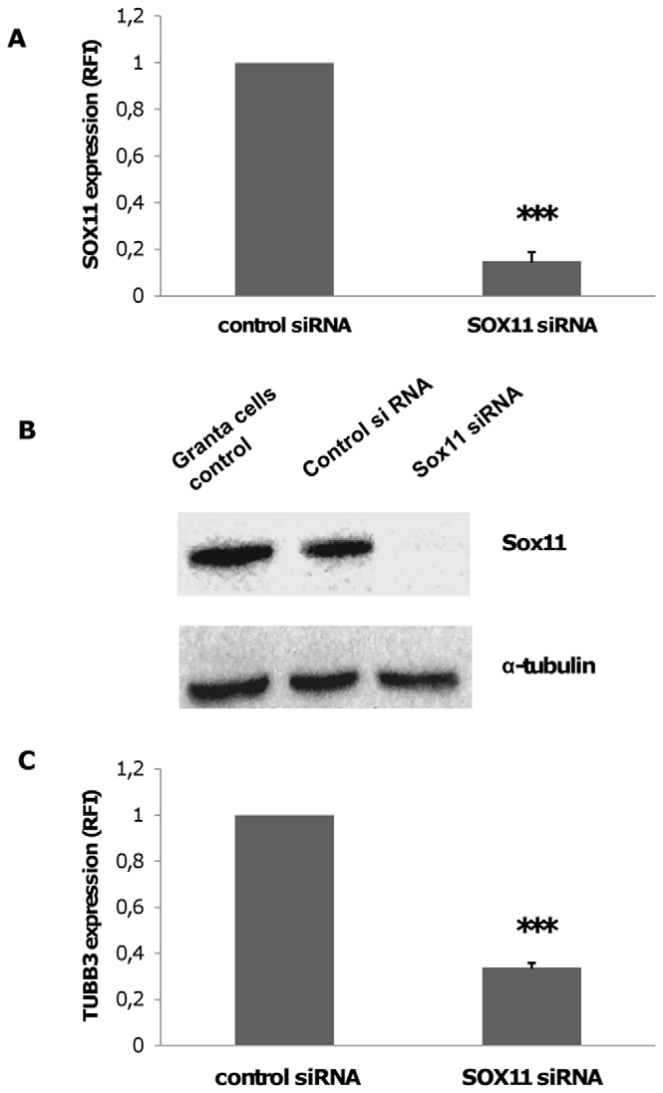
SOX11 silencing significantly reduces TUBB3 expression in the MCL cell line Granta 519. (A) Expression of SOX11 was evaluated after transfection with either 100 pmol SOX11 siRNA or control siRNA. The SOX11 mRNA level was measured by quantitative RT-PCR 20 hours after electroporation. Error bars show standard deviation of 4 independent experiments, ***p<0.001. (B) Western blots show downregulation of SOX11 protein in Granta 519 after SOX11 siRNA knockdown but not in cells treated with control siRNA or in untransfected cells. (C) Depletion of SOX11 protein significantly reduces TUBB3 expression. Granta 519 cells were transfected with SOX11 siRNA and TUBB3 expression was investigated by quantitative RT-PCR after 20 hours. ***P<0.001.

The effect of siRNA-inhibition of SOX11 in Granta 519 was investigated using the S13312 siRNA. After 20 hours, the expression of SOX11 mRNA decreased by approximately 85% compared to cells treated with control siRNA (Figure 2A). Downregulation of mRNA was stable between 12–48 hours (data not shown). The SOX11 protein could not be detected at 20 hours after SOX11 knock down (Figure 2B) and the protein levels remained undetectable for 72 hours (data not shown). Similar silencing effects were obtained by another SOX11 siRNA, S224667 (data not shown). TUBB3 has been shown to be regulated by Sox11 in non-hematopoietic cells [8]. We therefore investigated whether SOX11 is able to regulate TUBB3 in Granta 519. 20 hours after transfection with SOX11 siRNA the expression of TUBB3 was reduced by 70% compared to negative control siRNA (Figure 2C).

### Silencing of SOX11 Significantly Reduces the Expression of 26 Genes

We next analyzed the effect of knock down of SOX11 on global gene expression in Granta 519 cells. At the chosen time point, 20 hours after transfection, 26 genes were significantly downregulated in SOX11 siRNA treated cells compared to cells treated with control siRNA at the chosen threshold (FDR<0.002, FC≥1.5 or FC≤−1.5) (Table 1). No upregulated genes were found using this threshold.

**Table 1:** Differently expressed genes after SOX11 knockdown by siRNA in Granta 519 cells#.

probe set ID	gene_assignment	p-value*	Fold-Change*
8174189	NM_021992//TMSB15A//thymosin beta 15a//Xq21.33-q22.3	6.9064e-010	−3.94
8040070	NM_003108//SOX11//SRY (sex determining region Y)-box 11//2p25	4.3570e-009	−3.05
8110589	NM_015455//CNOT6//CCR4-NOT transcription complex, subunit 6//5q35.3	7.7295e-009	−1.52
7963054	NM_006009//TUBA1A//tubulin, alpha 1a//12q12-q14	2.2166e-008	−2.04
8117640	NM_025231//ZSCAN16//zinc finger and SCAN domain containing 16//6p22.1	3.8797e-008	−1.55
8047127	NM_001130158//MYO1B//myosin IB//2q12-q34	4.4570e-008	−2.43
8113504	NM_004772//C5orf13//chromosome 5 open reading frame 13//5q22.1	1.6184e-007	−1.78
8077370	NM_006515//SETMAR//SET domain and mariner transposase fusion gene//3p26.1	2.4819e-007	−1.58
8069880	NM_003253//TIAM1//T-cell lymphoma invasion and metastasis 1//21q22.1|21q22	2.6924e-007	−1.71
8169073	NM_194324//TMSB15B//thymosin beta 15B//Xq22.2	2.7046e-007	−1.70
7925550	NM_001126//ADSS//adenylosuccinate synthase//1cen-q12	2.7195e-007	−1.72
8005458	NM_001040078//LGALS9C//lectin, galactoside-binding, soluble, 9C//17p11.2/	4.1908e-007	−1.69
8170468	NM_005342//HMGB3//high-mobility group box 3//Xq28	4.9152e-007	−1.53
8013450	NM_001042685//LGALS9B//lectin, galactoside-binding, soluble, 9B//17p11.2	8.6597e-007	−1.71
8177222	NM_013230//CD24//CD24 molecule//6q21	9.3981e-007	−2.20
8102311	NM_001226//CASP6//caspase 6, apoptosis-related cysteine peptidase//4q25	1.1825e-006	−1.58
8091103	NM_006286//TFDP2//transcription factor Dp-2 (E2F dimerization partner 2)	1.2042e-006	−1.61
7930882	NM_207009//FAM45A//family with sequence similarity 45, member A//10q25	1.2641e-006	−1.51
8127109	NM_016513//ICK//intestinal cell (MAK-like) kinase//6p12.1	3.0653e-006	−1.67
8116051	NM_080881//DBN1//drebrin 1//5q35.3	3.4139e-006	−1.71
8005809	NM_009587//LGALS9//lectin, galactoside-binding, soluble, 9//17q11.2//396	3.8368e-006	−1.86
8135915	NM_013332//C7orf68//chromosome 7 open reading frame 68//7q32.1	4.6540e-006	−2.24
8150186	NM_024787//RNF122//ring finger protein 122//8p12	6.2295e-006	−1.85
8075263	NM_003634//NIPSNAP1//nipsnap homolog 1 (C. elegans)//22q12.2	6.4654e-006	−1.56
8074780	NM_013313//YPEL1//yippee-like 1 (Drosophila)//22q11.2	2.6161e-005	−1.99
7930181	NM_020682//AS3MT//arsenic (+3 oxidation state) methyltransferase//10q24.32	0.0001	−1.56

# Gene expression levels were assayed using the Affymetrix GeneChip Human Gene 1.0 ST Array.

*SOX11 siRNA vs. negative control siRNA transfected Granta 519 cells. Data analysis was done using the Partek Genomics suite software at a stringent threshold (FDR<0.002, fold changes ≤−1.5 or ≥1.5).

### SOX11 Expression Correlates to DBN1, SETMAR and HIG2 Expression in Two Independent MCL Cohorts

To test the generality of our findings we investigated how the expression of the 26 downregulated genes correlated to SOX11 expression in 16 primary MCL, all positive for cyclin D1 and SOX11 [4]. In addition to SOX11, nine of the 26 genes were represented on the U133A chip used in this analysis. Of these, HIG2, SETMAR and DBN1 were significantly correlated to SOX11 expression values (Table 2). During the course of our study, Fernandez et al. provided evidence for lack of SOX11 expression in leukemic MCL with indolent clinical course [19]. We therefore investigated possible correlation with SOX11 among these MCL cases (GSE 16455). 19 of our 26 genes defined by SOX11siRNA were represented in this data set. The expression of 15 genes of these genes was significantly correlated to SOX11 expression, including DBN1, SETMAR and HIG2 (Table 3).

**Table 2:** Validation of SOX11 siRNA downregulated genes in a dataset of 16 primary MCLa).

Probe set	Gene symbol	Spearman ratio#	p-value#
204915_s_at ***	SOX11	1.00	0.00
206554_x_at *	SETMAR	0.56	0.02
218507_at *	HIG2	0.57	0.02
202806_at *	DBN1	0.52	0.03
213996_at	YPEL1	0.44	0.08
219897_at	RNF122	−0.37	0.16
208650_s_at	CD24	−0.34	0.20
208651_x_at	CD24	0.13	0.63
209771_x_at	CD24	−0.12	0.66
209772_s_at	CD24	0.03	0.91
216379_x_at	CD24	−0.07	0.78
203236_s_at	LGALS9	0.19	0.48
201709_s_at	NIPSNAP1	−0.16	0.55
201708_s_at	NIPSNAP1	0.11	0.70
204569_at	ICK	−0.11	0.68

a) Data from reference (51) representing 16 SOX11+ MCL.

# The values were first tested for normal distribution. At the 0.05 level, all SOX11 data were not drawn from a normally distributed population, so we used Spearman rank correlation coefficient. The Spearman ratio represents the correlation between the values from SOX11 probe set 204915_s_at and the probesets of the selected genes. 2-tailed test of significance was used.

*Moderate correlation (correlation coefficient 0.4–0.7);

***Very high correlation (correlation coefficient 0.9–1.0).

**Table 3:** Validation of SOX11 siRNA downregulated genes in a series of SOX11+ and SOX11- MCLa).

probeset	gene symbol	Spearman ratio #	p value#
204915_s_at***	SOX11	1	0
204914_s_at***	SOX11	0.95	1.35E-11
204913_s_at***	SOX11	0.93	4.46E-10
201310_s_at **	C5orf13	0.84	1.33E-06
201309_x_at **	C5orf13	0.78	1.97E-05
230424_at **	C5orf13	0.78	2.16E-05
238411_x_at	C5orf13	0.29	0.18
203744_at **	HMGB3	0.82	3.10E-06
225601_at **	HMGB3	0.79	1.23E-05
202806_at **	DBN1	0.81	4.12E-06
217025_s_at *	DBN1	0.67	6.06E-04
214051_at **	MGC39900	0.73	1.15E-04
206554_x_at *	SETMAR	0.71	1.95E-04
1554059_at*	SETMAR	0.45	0.03
1554060_s_at*	SETMAR	0.43	0.05
209790_s_at *	CASP6	0.70	2.55E-04
211464_x_at*	CASP6	0.46	0.03
209118_s_at *	TUBA1A	0.69	3.63E-04
226157_at*	TFDP2	0.62	0.00
203588_s_at*	TFDP2	0.62	0.00
203589_s_at*	TFDP2	0.51	0.01
217970_s_at *	CNOT6	0.50	0.02
222476_at	CNOT6	0.11	0.64
205347_s_at *	TMSB15A	0.46	0.03
209772_s_at*	CD24	0.46	0.03
208650_s_at*	CD24	0.44	0.04
208651_x_at	CD24	0.39	0.07
216379_x_at	CD24	0.36	0.10
209771_x_at	CD24	0.36	0.10
227310_at*	ADSS	0.46	0.03
221761_at	ADSS	-0.29	0.19
218507_at *	HIG2	0.44	0.04
1554452_a_at *	HIG2	0.44	0.04
213135_at *	TIAM1	0.43	0.04
206409_at	TIAM1	0.17	0.44
225351_at *	FAM45A	0.44	0.04
1563919_a_at	FAM45A	0.17	0.46
1563920_at	FAM45A	-0.06	0.79
204569_at	ICK	0.40	0.06
1552837_at	ICK	0.18	0.43
212364_at	MYO1B	-0.30	0.17
212365_at	MYO1B	-0.12	0.58
203236_s_at	LGALS9	0.11	0.64
219676_at	ZSCAN16	0.22	0.34

a) Data retrieved from (GSE15455) representing 7 indolent and 15 conventional MCL.

# The values were first tested for normal distribution. At the 0.05 level, all SOX11 data were not drawn from a normally distributed population, so we used Spearman rank correlation coefficient. The Spearman ratio represents the correlation between the values from SOX11 probe set 204915_s_at and the probesets of the selected genes. 2-tailed test of significance was used.

*Moderate correlation (correlation coefficient 0.4–0.7);

**High correlation (correlation coefficient 0.7–0.9);

***Very high correlation (correlation coefficient 0.9–1.0).

### Expression of SETMAR and DBN1 are Influenced by SOX11 *in vitro* and SOX11 Expression Correlated to DBN1 in Primary MCL Cells

**Figure 3:**
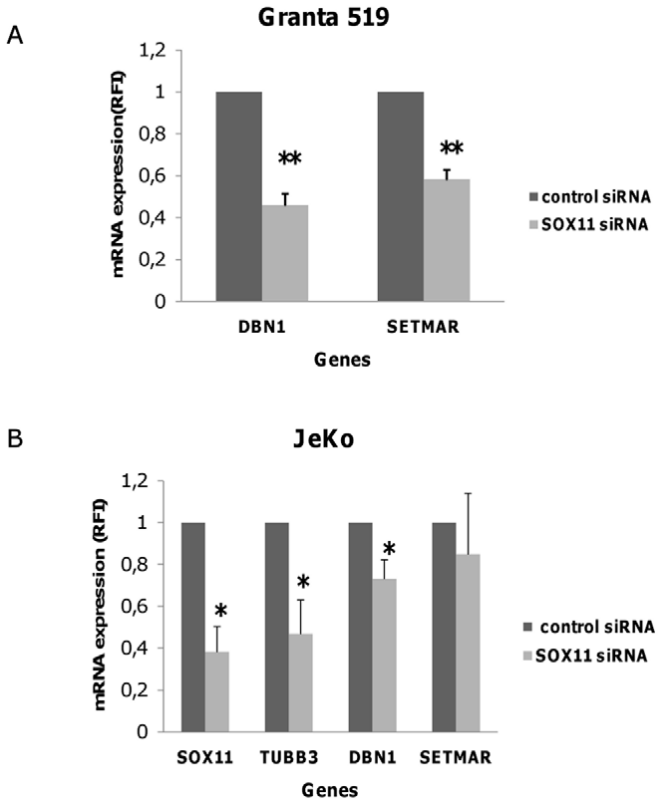
Reduced expression of SETMAR and DBN1 in response to SOX11siRNA. Analysis by qRT-PCR confirms downregulation of DBN1 and SETMAR (A) in SOX11 siRNA treated Granta 519 cells. (B) Expression of SOX11, TUBB3, SETMAR and DBN1 in the JeKo cell line was evaluated after transfection with either 100 pmol SOX11 siRNA or control siRNA. mRNA levels were measured by quantitative RT-PCR 20 hours after electroporation. Error bars show standard deviation of 4 independent experiments. * P<0.05, **P<0.01.

**Figure 4:**
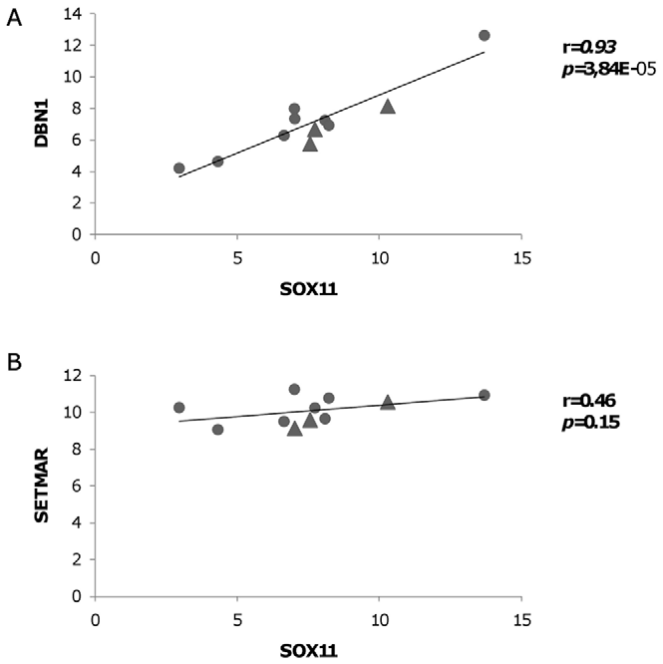
Expression of DBN1 correlates to SOX11 expression in primary MCL cells and MCL cell lines. The expression values of SOX11, DBN1 or SETMAR were analyzed by quantitative RT-PCR. The threshold cycle (Ct) of the gene against the Ct of β actin was determined by subtraction. All values were first tested for normal distribution, and then the Pearson correlation coefficients and P value were calculated. ▴ MCL cell lines Granta 519, Rec1 and JeKo; • primary MCL patients.

To validate the results of the gene expression analysis, SETMAR and DBN1 were analysed by quantitative RT-PCR in Granta 519 after SOX11 siRNA treatment. Both SETMAR and DBN1 mRNA levels were significantly (p<0.001) reduced by SOX11 downregulation in Granta 519 cells (Figure 3A). In another MCL cell line, JeKo, SOX11 downregulation caused similar reduction of TUBB3 and DBN1 levels but not of SETMAR (Figure 3B). We also found a significant correlation between the expression of SOX11 and DBN1 in primary MCL cells (8 samples from 6 patients as described in Materials and Methods and Table 4) and in the MCL cell lines Granta 519, Rec1 and JeKo (Figure 4).

**Table 4:** Isolated primary MCL cells from 6 patientsa).

Sample number	Patient age	sex	tissue	tumor cell content#
A1	82	M	blood	86%
A2	82	M	pleural exudate	95%
B1	77	F	blood	89%
B2	77	F	bone marrow	92%
C	69	M	spleen	80%
D	73	M	spleen	88%
E	77	M	lymph node	85%
F	66	M	blood	69%

a) In two patients (A and B) tumor cells from different tissues were retrieved and analyzed separately.

# Percentage of tumor cells as determined by flow cytometry.

### DBN1, SETMAR, and HIG2 are Direct Targets of SOX11 Protein

**Figure 5:**
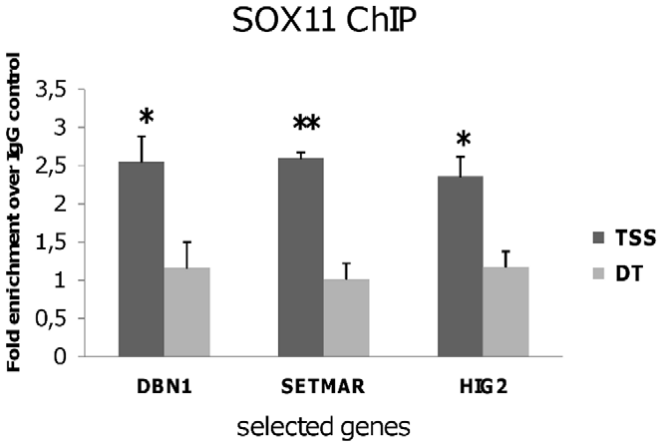
SOX11 protein directly targets DBN1, SETMAR and HIG2. ChIP assay of SOX11 protein binding to the selected three genes. Immunoprecipitated DNA was analyzed by quantitative RT-PCR for two different primers as described in material and method. TSS: primer designed close to transcription start site (TSS) and DT: primer distal (∼2 kb) to the TSS. Columns represent the mean of fold enrichment of DBN1, SETMAR or HIG2 relative to IgG control. Error bars show standard deviation of 3 independent experiments.* P<0.05, ** P<0.01 compared to the IgG control.

To investigate whether DBN1, SETMAR and HIG2 are direct targets of SOX11 protein, we performed chromatin immunoprecipitation (ChIP) assays on Granta 519 cells using two pairs of primers for each gene as described in Material and Methods. Granta 519 cells were cross-linked, and protein-DNA complexes were immunoprecipitated using antibodies recognizing normal rabbit IgG or anti-SOX11 antibody. We found that SOX11 was significantly recruited to DBN1, SETMAR and HIG2 promoter regions close to the transcription start site (TSS), but not to regions 2 kb distal to the TSS (DT) (Figure 5).

## Discussion

The main finding of this study is the identification of 26 genes influenced by SOX11 in MCL. SOX11 has critical roles in embryonic neurogenesis and tissue remodeling [5], [9], [23], [24]. It is also required for neuron survival and neurite growth [25] but no defined role in hematopoiesis has yet been shown.

The SOX11 protein belongs to the HMG box super family of DNA-binding proteins which are highly conserved between animals. They are involved in the regulation of such diverse development processes as specification of early embryonic germ layers [26], [27], organ development and neurogenesis [28]. SOX11, together with two other SOX family members SOX4 and SOX12, belong to the same subgroup, SOXC. SOX4 is so far the only gene in this family that has been found to have an important role in lymphopoiesis. SOX4–null hematopoietic cells grafted into wild type mice remain blocked at the pro-B cell stage [12]. Although SOX11 is highly homologous to SOX4, its role in lymphopoiesis remains unclear.

Recently, SOX11 was found to be aberrantly expressed in MCL [1], [2], [3], [4], [18]. Importantly, approximately 10% of cyclin D1 positive MCL lack nuclear expression of SOX11 [2], [4], [19]. SOX11 is also expressed in subsets of Burkitt lymphomas, lymphoblastic leukemias and hairy cell leukemias [1], [3], [18]. Thus, expression of SOX11 in lymphoma seems independent of the t(11;14) cyclin D1 translocation.

The possible role of the aberrant SOX11 expression in lymphoma is unknown. Others have shown that SOX11 is needed for the expression of pan-neuronal genes, including the Class-Ш β-tubulin gene TUBB3 (also named *Tuj1*). TUBB3 was the first gene to be identified as a potential direct target of SOXC proteins. In this study, we showed that SOX11 can modify the expression of TUBB3 also in MCL.

By gene expression analysis using a very stringent threshold, we identified 26 genes, all significantly downregulated, after SOX11 knockdown in MCL cells. Three of these genes were also found to be significantly correlated to SOX11 expression levels in 16 SOX11 positive primary MCL samples from various tissues. During the course of our study, Fernandez et al. published results on MCL with leukemic, non-nodal presentation and a very indolent clinical course [19]. Many of these tumors had in fact been misdiagnosed as other types of lymphomas. 15 of the 26 SOX11siRNA downregulated genes in Granta 519 were found to be significantly correlated to SOX11 expression in the Fernandez cohort, confirming our observations.

Prior to this study, Tubb3 and Tead2 were the only genes proven to be directly regulated by Sox11 [8], [9]. By chromatin immunoprecipitation we showed direct binding of the SOX11 protein to the promoter regions of the DBN1, SETMAR and HIG2 genes in MCL cells.

DBN1 and three other genes downregulated by SOX11 silencing, TMSB15A, TMSB15B and C5orf13 are actin binding and involved in cell shape and motility. DBN1 encodes for the actin binding protein drebrin 1 (*d*evelopmentally *re*gulated *br*ain protei*n*), cloned in 1988 [29], [30]. DBN1 was later shown to be is involved in neurite formation and synaptic signalling (reviewed in [31]) and to be downregulated in brains of Alzheimer patients [32], [33]. Drebrin is also expressed in epithelial cells of the stomach, kidney, colon and in cell lines from fibroblasts and astrocytoma [34]. Interestingly, drebrin is cleaved by caspase-6 [35], encoded by another of the SOX11siRNA downregulated genes.TMSB15A and TMSB15B encode for different isoforms of the actin binding protein thymosin beta 15. Thymosin beta 15 promotes cell motility and was discovered as highly upregulated in human breast, prostate and colon cancer [36], [37], [38], [39]. According to NCBI, C5orf13 is coding for the protein P311, highly expressed in glioma and involved in glioma cell migration through the reorganization of the actin cytoskeleton [40]. Thus a number of genes involved in cell shape and motility were downregulated by SOX11 siRNA treatment of Granta 519 cells. We could, however, not detect any significant change in shape or growth pattern of the cells after siRNA treatment (data not shown).

SETMAR, HIG2 and HMGB3 could potentially influence cell division and response to cytostatic treatment. SETMAR, also called METNASE, is cooperating with topoisomerase II alpha in decoiling of chromosomes during mitosis. Recent evidence in acute myeloid leukemia suggests that SETMAR may confer resistance to the topoisomerase II alpha inhibitor VP-16 [41], [42]. The HMGB3 protein belongs to the high mobility group of proteins and may, as shown for other HMG proteins, play a role in DNA replication and transcription. Hmgb3 is highly expressed in hematopoietic stem cells [43]. Recent experiment in mice indicate that Hmgb3 downregulation is associated with increased Wnt-signalling, more rapid renewal of stem cells and fewer lymphoid and myeloid progenitor cells [44]. We therefore investigated a possible influence on cell proliferation or cell survival *in vitro* after SOX11 knock-down by siRNA. In these short term experiments, we did however not detect any significant changes in cell proliferation or viability after SOX11 downregulation (manuscript in preparation), in contrast to what has been reported for neural cells [25]. However, since the effect on gene expression in siRNA treated cells is transient, we cannot exclude possible effects of SOX11 expression on cell survival or proliferation in other experimental settings.

SOX11 is aberrantly expressed in MCL but the molecular mechanism(s) responsible for its upregulation in lymphoid and hematopoietic cells are not yet defined, in contrast to other cell types. In chicken neural cells overexpression of Ngn2 and Ascl1 upregulates Sox11 while the transcriptional repressors Id1 and REST/NRSF downregulates its expression [11]. In the mouse embryonal neuronal plate Sox11 was upregulated by FoxD5 and Notch signalling [45], [46]. SOX11 mRNA expression was also rapidly (within 3 hours) upregulated after exposure of retinal microglia to 17beta-estradiol [47]. Even though SOX11 is highly expressed in most MCL, expression of SOX11 seems not to be dependent on the t(11;14) since it is a feature of both cyclin D1 positive and cyclin D1 negative cases [3]. Furthermore, SOX11 may be expressed in other aggressive B cell lymphomas that lack cyclin D1.

In summary, we have used selective siRNA targeting and downregulation of SOX11 to identify a set of SOX11 responsive genes. Our results have been validated in primary MCL tumors and freshly isolated lymphoma cells. Three of the identified genes, DBN1, SETMAR and HIG2, were found to be directly targeted by the SOX11 protein in MCL.

## Materials and Methods

### Ethical Permission

This study was approved by the Ethical Committe at the Karolinska Institutet, and performed according to the principles expressed in the Declaration of Helsinki. All the patients gave their written informed consent to participate in the study.

### Patient Samples

Eight samples of tumor cells were collected from 6 patients diagnosed with cyclin D1+ MCL at the Department of Pathology (Karolinska University Hospital Huddinge, Sweden) (Table 4). Tumor cells were isolated from different tissues (bone marrow, blood, lymph node, spleen, pleural exudate), viability frozen in RPMI 1640 medium containing 10% DMSO and 40% FBS and stored at -135°C.

### Cell Lines

Granta 519, Rec1, JeKo, JVM2 cell lines and primary cells were obtained and cultured as previously described [48]. In short, cell lines were maintained in culture medium (RPMI 1640, supplemented with 10% fetal bovine serum (FBS) and 50 µg/ml gentamicin (all from Invitrogen, Carlsbad, CA)) under standard conditions (humidified atmosphere, 95% air, 5% CO_2_, 37°C). The cells were maintained between 3×10^5^ and 1.5×10^6^/ml. Culture medium was changed twice weekly.

### siRNA and Transient Transfection

Two silence ® select pre-designed SOX11 siRNAs, S13312 and S224667, and one non-targeting silence® select Negative Control #1 siRNA were obtained from Ambion (Ambion, Austin, TX). siRNA S13312, used in most experiments had the sense targeting sequence (5′-3′): GACCUGAUGUUCGACCUGAtt. Results were confirmed using S224667 with the sense targeting sequence (5′-3′): GGAGCUGAGCGAGAUGAUCtt. Freeze-dried siRNAs were suspended in nuclease-free water (Ambion, Applied Biosystem, Austin, TX) in a final concentration of 50 µM. Transient transfection were performed on an Amaxa Nucleofection Device (Lonza Cologne AG, Cologne, Germany) according to the manufacturer's instruction. In brief, Granta 519 cells were split at a density of 5×10^5^/ml in the medium 48 hours before transfection. Thereafter, 4×10^6^ cells were collected and resuspended in 100 µl human cell line nucleofector solution C with 100 pmol of either SOX11 siRNA or control siRNA using the X-01 electroporation program. The two SOX11 siRNAs were tested for their individual ability to knock down SOX11 expression. Both siRNA sequences were able to knock down SOX11 expression by 80%-85% in Granta 519 cells.

### RNA Isolation and Affymetrix GeneChip Human Gene 1.0 ST Array

Cells were harvested 20 hours after siRNA transfection. Total RNA from individual transfection experiments in Granta 519 cells (n = 4) or patient samples (n = 8) was extracted with Qiagen RNeasy Plus Mini kit according to the instructions of the manufacturer (Qiagen, Valencia, CA). RNA quantity was measured using a NanoDrop (NanoDrop Technologies, Wilmington, DE) set for RNA measurement (A260/A280 ratio). RNA quality was assessed using an Agilent Bioanalyzer 2100 (Agilent Technologies, Palo Alto, CA) and samples with high-quality RNA were hybridized to Affymetrix GeneChip Human Gene 1.0 ST arrays (Affymetrix Inc. Santa Clara, CA) in accordance with the Affymetrix standard protocol at the Bioinformatics and Expression Analysis Core facility (BEA, Department of Biosciences and Nutrition, Karolinska Institutet).

### Gene Expression Data Analysis

The microarray image data were processed in the BEA core facility according to standard procedures. CEL data was analyzed with Partek Genomic Suite vs 6.5 (Partek Inc., St. Louis, MO) which can normalize and process multiple datasets simultaneously. Data normalization was performed by Robust Multiarray Analysis algorithm (RMA). Statistical differences were examined using one way ANOVA. Significant probesets were defined with a p-value cutoff significant with a False Discovery Rate (FDR) of <0.002, and a fold-change (FC) equal or greater than 1.5(FC≥1.5) for upregulated genes and equal or less than −1.5(FC≤1.5) for downregulated genes, respectively.

Gene expression values for the selected genes were retrieved from two primary array databases. Our previously published gene expression analysis included 16 MCL tumor samples, all expressing cyclin D1 and SOX11 [49]. We also retrieved data from a recently published study from [19] which included 7 indolent, SOX11 negative MCL and 15 conventional, SOX11 positive MCL (http://www.ncbi.nlm.nih.gov/geo/with the GSE accession number GSE16455).

### cDNA Synthesis and Real-time Quantitative RT-PCR

cDNA was generated according to the protocol for Omniscript Reverse Transcription (Qiagen, GmbH, Hilden, Germany). 2 µg of RNA was used in conjunction with Oligo(dT) primer and 20 µl of cDNA was generated. 1 µl of cDNA was added to qPCR Kit Platinum SYBR Green qPCR SuperMix-UGD with FITC (Invitrogen) according to the manufacturer's instructions, run in triplicate on a CFX96 Real-time System (C1000 Thermal Cycler, BIO-RAD, city, CA). The following primers were used: SOX11, 5′-CATGTAGACTAATGCAGCCATTGG-3′ and 5′-CACGGAGCACGTGTCAATTG-3′; SOX4, 5′- CAGCCCCTAATTTCTCCATGTT and 5′- GGTGGCAGGTTAAGGGATACTG; SOX12, 5′- CCCAGGTCCACCCTCAGTAC and 5′- CGAGAGTCTTCCTGCCATCAC; SETMAR, 5′-GCGGAAGCGGCAAAGAC-3′ and 5′-GCCTCAGGCTTCTCCTTAAACTC-3′; DBN1, 5′-GCCCCACCTGCTAACCAA-3′ and 5′-GTGATTGACTGAAGTACCCCTCACT-3′; TUBB3,5′- GGAGCGGATCAGCGTCTACT-3′ and 5′-GCTCGAGGCACGTACTTGTG-3′; β-actin, 5′-AAAGACCTGTACGCCAACACA-3′ and 5′-AGTACTTGCGCTCAGGAGGA-3′. The cycle parameters used was: 50°C 2 min, 95°C 2 min, followed by 40 cycles of 95°C for 15 seconds and 59°C for 30 seconds. Ct values (Threshold cycles) were obtained from amplification of SOX11, SOX4, SOX12, SETMAR, DBN1, TUBB3 and β-actin. The ΔCt value was calculated by subtracting the Ct value of β-actin from the Ct value of target gene. A ΔΔCt value was then calculated by subtracting the ΔCt value for siRNA condition from the ΔCt value for control condition with relative fold increase (RFI) reported as 2^−ΔΔCt^.

### Western blotting

Western blotting was performed as described previously [4]. The blotted membranes were probed with rabbit anti-human SOX11 (HPA000536, Atlas Antibodies, Uppsala, Sweden), and antibodies to tubulin (SC 8035, Santa Cruz Biotechnology, Santa Cruz, CA) or actin (A4700, Sigma, Saint Louis, MO) was used as loading controls. Antibody binding was detected by enhanced chemoluminescence using SupersignalWest Pico (Pierce Biotechnology, Shelton) CTchemiluminescent substrate.

### Chromatin immunopreciption (ChIP)

Granta 519 cells were split at a density of 5×10^5^/ml in the medium and cultured for 72 hours. Thereafter, 2×10^7^ cells were collected and crosslinked with 1% formaldehyde for 10 min. Cross-linking was quenched by adding 125 mM glycine and cells were washed with cold PBS, harvested and resuspended in lysis buffer containing protease inhibitors cocktail (Roche, Mannheim, Germany) and sonicated 15 min for three times. The soluble chromatin was collected by centrifugation and the supernatants were incubated with 30 µl protein A/G Sepharose (50% slurry; GE Healthcare Bio-Sciences Corp., Uppsala, Sweden) under gentle agitation over night at 4°C. The supernatant was transferred to a new microcentrifuge tube, followed by immunoprecipitation with 1 µg of anti-SOX11 antibody and non-immune rabbit IgG (Santa Cruz Biotechnology, Santa Cruz, CA) as control at 4 C overnight. Protein A/G Sepharose (20 µl of a 50% slurry) was then added and incubated for 1.5–2 h. The pellets were successively washed with different buffer as previously described [50]. Protein-DNA crosslinks were reversed by overnight incubation at 65°C in 120 µl elution buffer [TE; 1% SDS]. DNA was purified using a PCR purification kit (QIAGEN, Valencia, CA) and eluted in 50 µl of elution buffer. The immunoprecipitated DNA was amplified by real-time PCR using Fast SYBR Green Master Mix (Applied Biosystems, Warrington,UK) Two pairs of primers were designed for DBN1, SETMAR and HIG2 with one primer designed close to transcription start site (TSS) and one distal (∼2 kb) to the TSS (DT).

The primer pairs used are as follows:

DBN1 TSS forward: 5′-TGAGGTGGAAGGATGTTTGCT-3′; Reverse: 5′-CGGCGGTAAGGGAGTCACT-3′.

DBN1 DT forward: 5′-CCCTGCCGTGGGAGTCT-3′; Reverse: 5′-TCCCAGAGGAGTCCCAAGTAGA-3′.

SETMAR TSS forward: 5′-GGGAGCCAGACCCAAAAAGT-3′; Reverse: 5′-TTCTCAGGAGTGGCCTGGAA-3′.

SETMAR DT forward: 5′-AGAAAGATACAGAGAAGGGACTACTTGAG-3′; Reverse: 5′-TTCTTGTATCTCCAGTGCCTTTACC-3′.

HIG2 TSS forward: 5′-TCACTCCAGAACACAATGACTCAA-3′; Reverse: 5′-CGCGGAGCTGTTTCCAAA-3′.

HIG2 DT forward: 5′-TTTCCAACTGGCATGACCTTT-3′; Reverse: 5′-GCACCTCTCCAACAATTCTTTCTC-3′.

### Statistical Analysis

Quantitative RT-PCR data were statistically analyzed using paired *t* -test with P values <0.05 as statistical significance. The correlation between SOX11 and other genes as well as the expression differences among MCL were analyzed with the Spearman's rank correlation coefficient test or Pearson correlation coefficient for qRT-PCR results using Origin 8 software (OriginLab, MA USA) using Origin 8 software. 2-tailed test for significance was used.
